# Supplementary material for: Complex‐centric proteome profiling by SEC‐SWATH‐MS
Source: Mol Syst Biol. 2019 Jan 14;15(1):e8438. doi: 10.15252/msb.20188438 (PMC6346213; doi:10.15252/msb.20188438)
Supplement: Supplementary file 7 — Dataset EV6 [file MSB-15-e8438-s007.zip › feature_plots_bioplex/P51674.pdf]

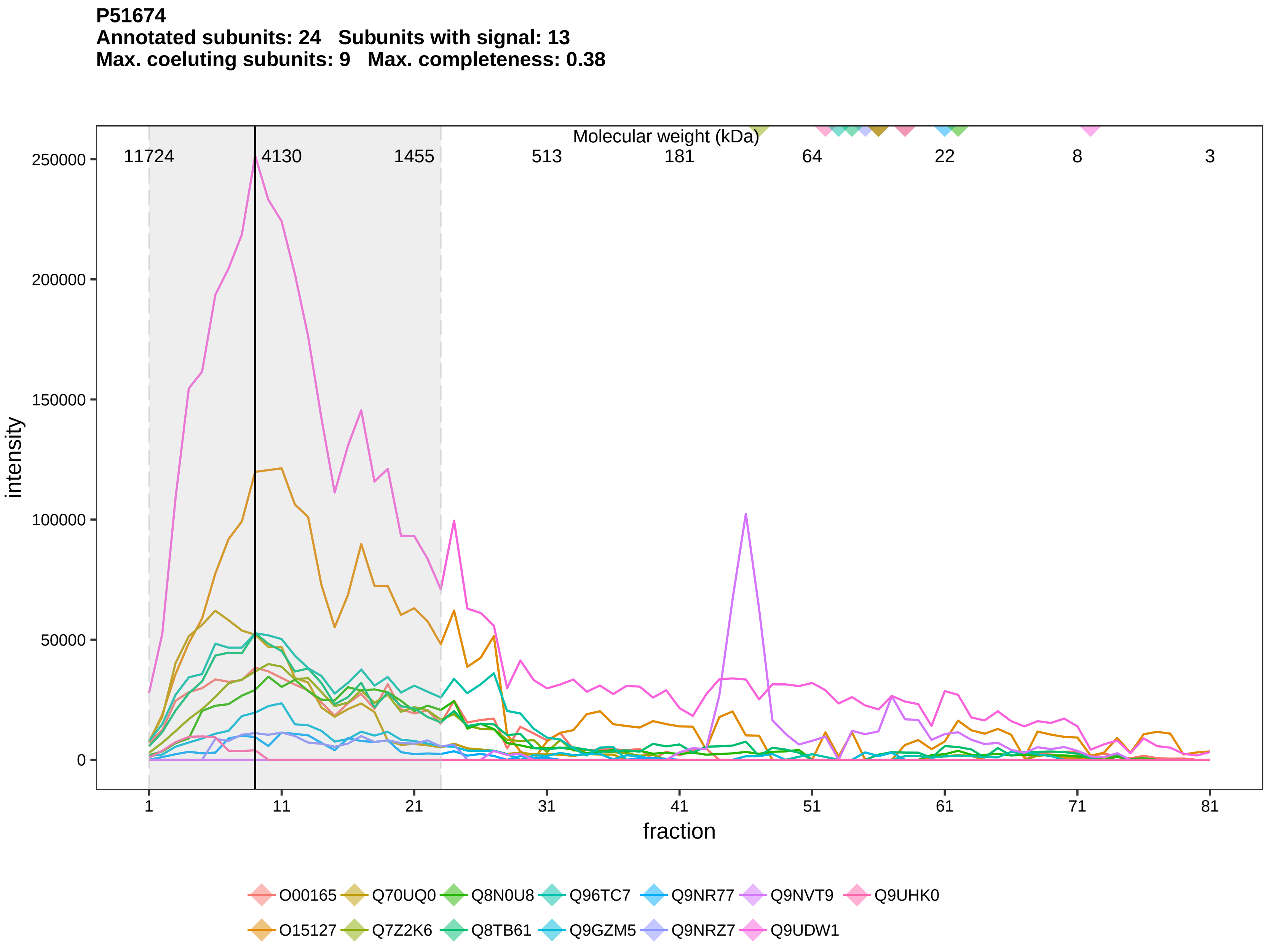
P51674
Annotated subunits: 24 Subunits with signal: 13
Max. coeluting subunits: 9 Max. completeness: 0.38
Molecular weight (kDa)
11724
4130
1455
513
181
64
22
8
3
250000
200000
150000
100000
50000
0
intensity
1
11
21
31
41
51
61
71
81
fraction
O00165
Q70UQ0
Q8N0U8
Q96TC7
Q9NR77
Q9NVT9
Q9UHK0
O15127
Q7Z2K6
Q8TB61
Q9GZM5
Q9NRZ7
Q9UDW1
